# Electrocorticographic Activation Patterns of Electroencephalographic Microstates

**DOI:** 10.1007/s10548-023-00952-1

**Published:** 2023-03-20

**Authors:** Christian A. Mikutta, Robert T. Knight, Daniela Sammler, Thomas J. Müller, Thomas Koenig

**Affiliations:** 1 Translational Research Center, University Hospital of Psychiatry and Psychotherapy, University of Bern, Bern, Switzerland; 2 Private Clinic Meiringen, Meiringen, Switzerland; 3 Helen Wills Neuroscience Institute, University of California-Berkeley, 132 Barker Hall, 94720 Berkeley, CA USA; 4 Research Group Neurocognition of Music and Language, Max Planck Institute for Empirical Aesthetics, Frankfurt am Main, Germany; 5 Interdisciplinary Biosciences Doctoral Training Partnership, Department of Physiology, Anatomy and Genetics, University of Oxford, Oxford, UK

**Keywords:** Electrocorticography, Electroencephalography, Microstates, Resting state, Stereotactic electroencephalography

## Abstract

Electroencephalography (EEG) microstates are short successive periods of stable scalp field potentials representing spontaneous activation of brain resting-state networks. EEG microstates are assumed to mediate local activity patterns. To test this hypothesis, we correlated momentary global EEG microstate dynamics with the local temporo-spectral evolution of electrocorticography (ECoG) and stereotactic EEG (SEEG) depth electrode recordings. We hypothesized that these correlations involve the gamma band. We also hypothesized that the anatomical locations of these correlations would converge with those of previous studies using either combined functional magnetic resonance imaging (fMRI)-EEG or EEG source localization. We analyzed resting-state data (5 min) of simultaneous noninvasive scalp EEG and invasive ECoG and SEEG recordings of two participants. Data were recorded during the presurgical evaluation of pharmacoresistant epilepsy using subdural and intracranial electrodes. After standard preprocessing, we fitted a set of normative microstate template maps to the scalp EEG data. Using covariance mapping with EEG microstate timelines and ECoG/SEEG temporo-spectral evolutions as inputs, we identified systematic changes in the activation of ECoG/SEEG local field potentials in different frequency bands (theta, alpha, beta, and high-gamma) based on the presence of particular microstate classes. We found significant covariation of ECoG/SEEG spectral amplitudes with microstate timelines in all four frequency bands (p = 0.001, permutation test). The covariance patterns of the ECoG/SEEG electrodes during the different microstates of both participants were similar. To our knowledge, this is the first study to demonstrate distinct activation/deactivation patterns of frequency-domain ECoG local field potentials associated with simultaneous EEG microstates.

**Supplementary Information:**

The online version contains supplementary material available at 10.1007/s10548-023-00952-1.

## Introduction

Independent research involving electroencephalography (EEG), functional magnetic resonance imaging (fMRI), and magnetoencephalography (MEG) has led to the hypothesis that resting-state brain activity is a process of active self-organization, even in the absence of incoming stimuli (Fox and Raichle 2007; Jann, Kottlow, Dierks, Boesch, & Koenig, 2010; Michel & Koenig, 2017). In EEG research, microstate analysis has emerged as a reliable tool that can quantify resting-state network activity (Khanna et al. 2015). Because microstates are short successive periods of quasi-stable, large-scale scalp field potentials, they represent the spontaneous activation of brain resting-state networks (Schwab et al. 2015). Similar to the resting-state fMRI networks, a relatively small and replicable set of prototypical EEG microstate classes has been identified over the course of the past two decades; however, the number of distinguished states is typically smaller than that reported by fMRI studies.

It has been hypothesized that EEG microstates represent singular and global patterns of relatively slow and highly synchronized oscillations that regulate activity and gate the interactions among local brain structures to dynamically adapt the responsiveness of the brain to momentary environmental and internal conditions. Dysfunctional mental states such as schizophrenia and dementia (among many others) have typical alterations in particular classes of EEG microstates (Khanna et al. 2015; Koenig et al. 1999; Lehmann et al. 2005, 2014; Rieger, Diaz Hernandez, Baenninger, & Koenig, 2016; Smailovic et al., 2022; Tait et al., 2020). The mechanism by which this adaptive regulation is implemented has been assumed to be based on transient low-frequency fluctuations of cortical excitability that are globally organized (Communication through Coherence [CTC]) (Fries 2005), and that regulate the activity and interactions among local brain structures that predominantly occur in higher-frequency (broadband high-gamma) ranges (Leszczynski et al. 2020). EEG microstates and local, predominantly high-frequency activity are thus (under the assumptions of the current understanding) systematically interacting but distinct phenomena; the microstates are supposed to inform about the overall cognitive mode, whereas local activity is assumed to contain the specific representational contents (Michel and Koenig 2017). However, a direct link between EEG microstates and local changes in broadband high-gamma cortical oscillations has not yet been established (Lehmann et al. 2006; Michel and Koenig 2017). Studies that investigated local brain activity related to EEG microstates that used combined EEG and fMRI showed that there were consistent patterns of local changes in activation that were related to particular global microstate patterns (Britz et al. 2010; Rajkumar, Farrher, et al., 2021; Rajkumar, Regio Brambilla, et al., 2021); however, they lacked a representation in frequency effects because of the low temporal resolution of fMRI. Similarly, studies that used EEG inverse solutions identified converging localizations of EEG microstates (Custo et al. 2017); however, they did not address the issue that the assumed local changes in activity associated with those microstates may be distinguished by resolving the data in the frequency domain. This limitation is also attributable to the fact that more focal patterns of cortical activity are suppressed in scalp EEG because the EEG lead field acts as a spatial low-pass filter (Iivanainen et al. 2021).

We addressed this knowledge gap by combining a scalp EEG microstate analysis with electrocorticography (ECoG) and stereotactic EEG (SEEG), which are designed to specifically capture local brain activity. Intracranial recordings of local field potentials offer excellent spatial and temporal resolution. Furthermore, ECoG and SEEG recordings can improve the measurements of local broadband high-gamma activity to a degree that justifies their application in clinical conditions, such as epilepsy, despite the considerable risks they entail. In particular, broadband high-gamma frequency was found to provide excellent spatial discrimination (Canolty et al. 2007). Additionally, broadband high-gamma frequency was found to be a general index of cortical processing (Crone et al. 2011; Crone, Sinai, & Korzeniewska, 2006). Therefore, we hypothesized that transient and local changes in broadband high-gamma activity are consistently associated with the presence or absence of particular EEG microstate classes.

Based on the CTC theory, we proposed the following two hypotheses: (1) global scalp EEG-based microstate patterns are correlated with local field potential activation patterns in intracranial locations, and these correlations are frequency-dependent; and (2) the anatomical locations of these correlations would converge with those of previous studies using either combined fMRI-EEG (Mulert 2013; Rajkumar, Farrher, et al., 2021; Rajkumar, Regio Brambilla, et al., 2021; Rosenkranz & Lemieux, 2010) or EEG source localization (Custo et al. 2017; Milz, Pascual-Marqui, Achermann, Kochi, & Faber, 2017).

## Materials and Methods

### Participants

**Fig. 1:**
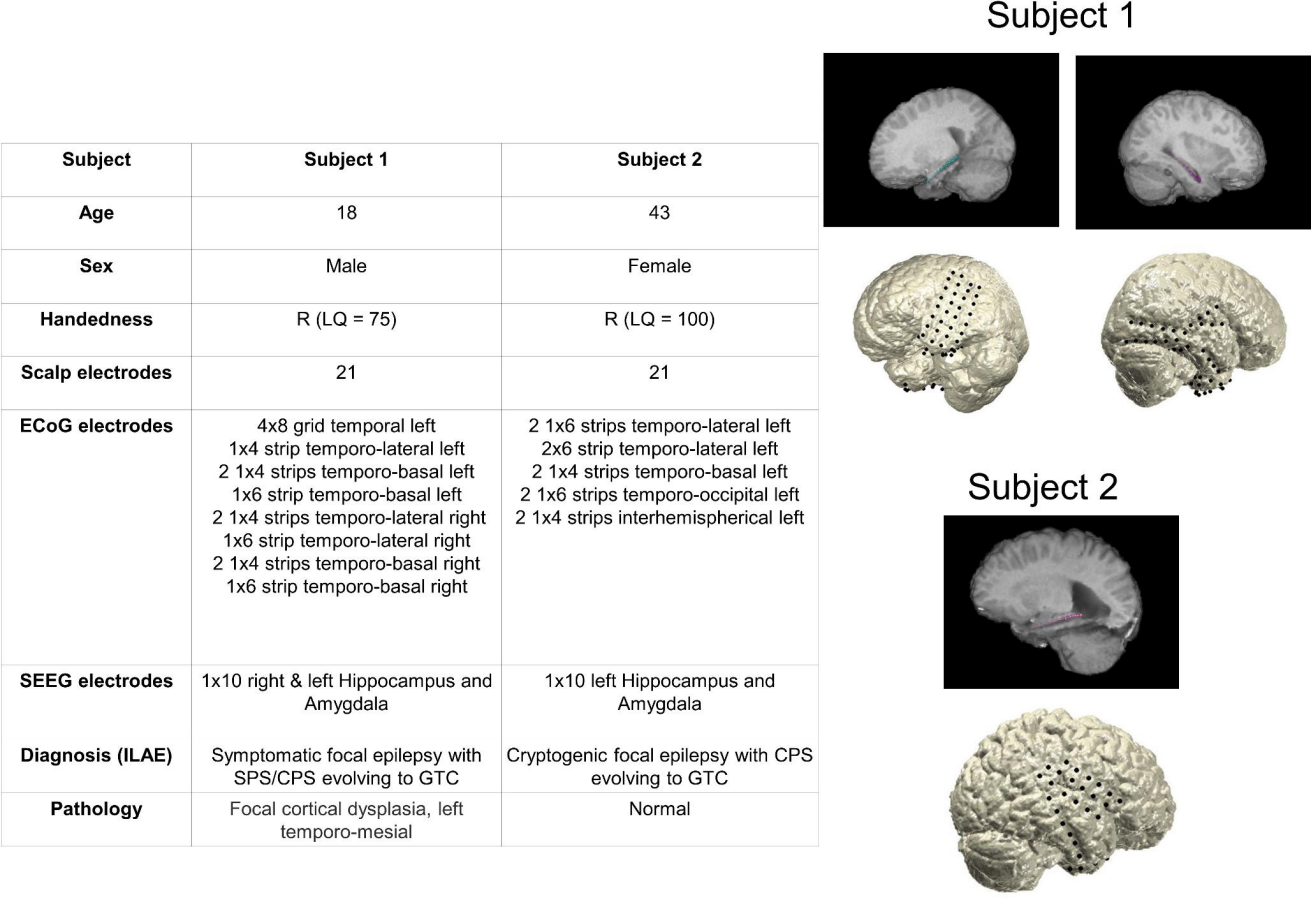
Magnetic resonance imaging (MRI) results and electrode positions of the two participants. The stereotactic electroencephalography (SEEG) depth electrode (10 contacts) covering the left and right hippocampi and amygdala of participant 1 is shown (upper panel). The electrocorticography (ECoG) grids covering the left and right temporal lobes of participant 1 are also shown. The depth electrode (10 contacts) covering the left amygdala and hippocampus of participant 2 is shown. The ECoG electrodes covered the right parietal, frontal, and temporal regions of the brain. Abbreviations: R: right, LQ:laterality quotient, ILAE: INternational League against Epilepsy diagnosis, CPS: complex partial seizures SPS: simple partial seizures; GTC: generalized tonic-clonic seizure.

Data were obtained from two adult participants who underwent a presurgical evaluation of pharmacoresistant epilepsy using subdural and stereotactic depth electrodes. The electrode placement was dictated exclusively by clinical considerations, and both participants provided written informed consent to participate in the study. Figure 1 summarizes the patient information, electrode coverage, and specific epilepsy diagnoses. The participants were native speakers of German, were right-hand-dominant, and had left-hemispheric language dominance. This study was conducted at the University Clinic of Freiburg (Freiburg, Germany) and was approved by the Ethics Committee of the University of Freiburg, Germany.

### EEG, ECoG, and SEEG Recordings and Data Reduction

Data were collected at the epilepsy center of the University Medical Center (Freiburg, Germany). The EEG signal was recorded from subdural grid and strip electrodes, as well as from depth electrodes placed according to medical needs. EEG, ECoG, and SEEG data were acquired using a Neurofile NT digital video EEG system (Natus Medical Inc., San Carlos, CA, USA) at 1024 Hz. We used 5-min resting-state recordings.

### Electrode Localization

For both participants, electrode locations were determined using co-registered and normalized preoperative and postoperative magnetic resonance imaging (MRI) with a resolution of 1 × 1 × 1 mm and the automated visualization method of Stolk et al. (Stolk et al. 2018), which is implemented as part of the FieldTrip toolbox (Oostenveld et al. 2011). Briefly summarized here: The preimplant MRI results were transformed into the Talairach space. Then, we segmented the MRI results using FreeSurfer 5.3.0. The three-dimensional electrode coordinates were determined using the automated visualization method described by Kovalev et al. (2005). The aligned electrodes were warped onto a template brain in the Montreal Neurological Institute (MNI) space to facilitate visualization. MNI reconstruction was performed only for visualization purposes, but electrode localization was determined in the native space. Figure 1 provides an overview of both participants’ ECoG and SEEG electrode positions. Scalp EEG electrodes were placed according to the traditional 10–20 system.

### Interictal Epileptic Discharge Detection

Before all the microstate analyses were performed, we first detected the interictal epileptic discharges (IEDs) of all channels using an established automated algorithm (Helfrich et al. 2019). The continuous EEG/ECoG/SEEG signal was filtered between 25 and 80 Hz (second-order finite impulse response), and the analytical amplitude was extracted using the Hilbert transform in MATLAB. Furthermore, the *z*-scores of the data were determined. The IED events were defined as a signal of three standard deviations above the mean for more than 20 ms and less than 100 ms. Periods of 2.5 s before and after the events comprised the artifact period.

### Preprocessing

The EEG, ECoG, and SEEG data were analyzed using Brain Vision Analyzer version 2.2. Eye movements in the EEG channels were corrected using an independent component analysis (Delorme, 2007). EEG channels with excessive artifacts were interpolated.

The ECoG grid electrodes were demeaned and notch-filtered at 50 Hz, and a within-grid common average reference was applied. The ECoG stripes and SEEG were referenced to their immediate lateral neighboring electrode (bipolar montage) and downsampled to 500 Hz. The scalp-EEG data was band-pass filtered between 2 and 20 Hz and recomputed to common average reference.

### Microstate Analysis

The Global Field Power (GFP) of the EEG of the participants, which quantifies the overall potential variance across a set of electrodes, was computed for each sample over time. Then, the topographies at the peaks of the GFP were assigned to the best-fitting template of the seven microstate templates reported by Custo et al.(Custo et al. 2017). For this purpose, these templates were spatially resampled to the 10–20 system using spherical spline interpolation. These template maps were chosen because they were based on a large normative sample, and their inverse solutions have been published. Based on this assignment of moments of momentary GFP peaks, the assignment of the remaining data was interpolated using a nearest neighbor interpolation. This yielded a timeline for each microstate class that indicated the amount of EEG variance explained by the microstate model of the given microstate class (zero when the data were not assigned to a given class). Then, these timelines were used as regressors for the SEEG and ECoG data in the covariance analysis.

### Covariance Mapping

To estimate the transient activation/deactivation of the ECoG and SEEG local field potentials in different frequency bands, the temporospatial evolution (Thut et al. 2006) was computed: First, the data were bandpass-filtered using different frequency bands (theta: 4–8 Hz; alpha: 9–12 Hz; beta: 13–30 Hz; low-gamma: 30–70 Hz; and broadband high-gamma: 70–150 Hz) using IIR bandpass filters. Then, the data were rectified, low-pass-filtered at a cutoff of half of the low cutoff frequency of the given band, and log-transformed.

**Fig. 2:**
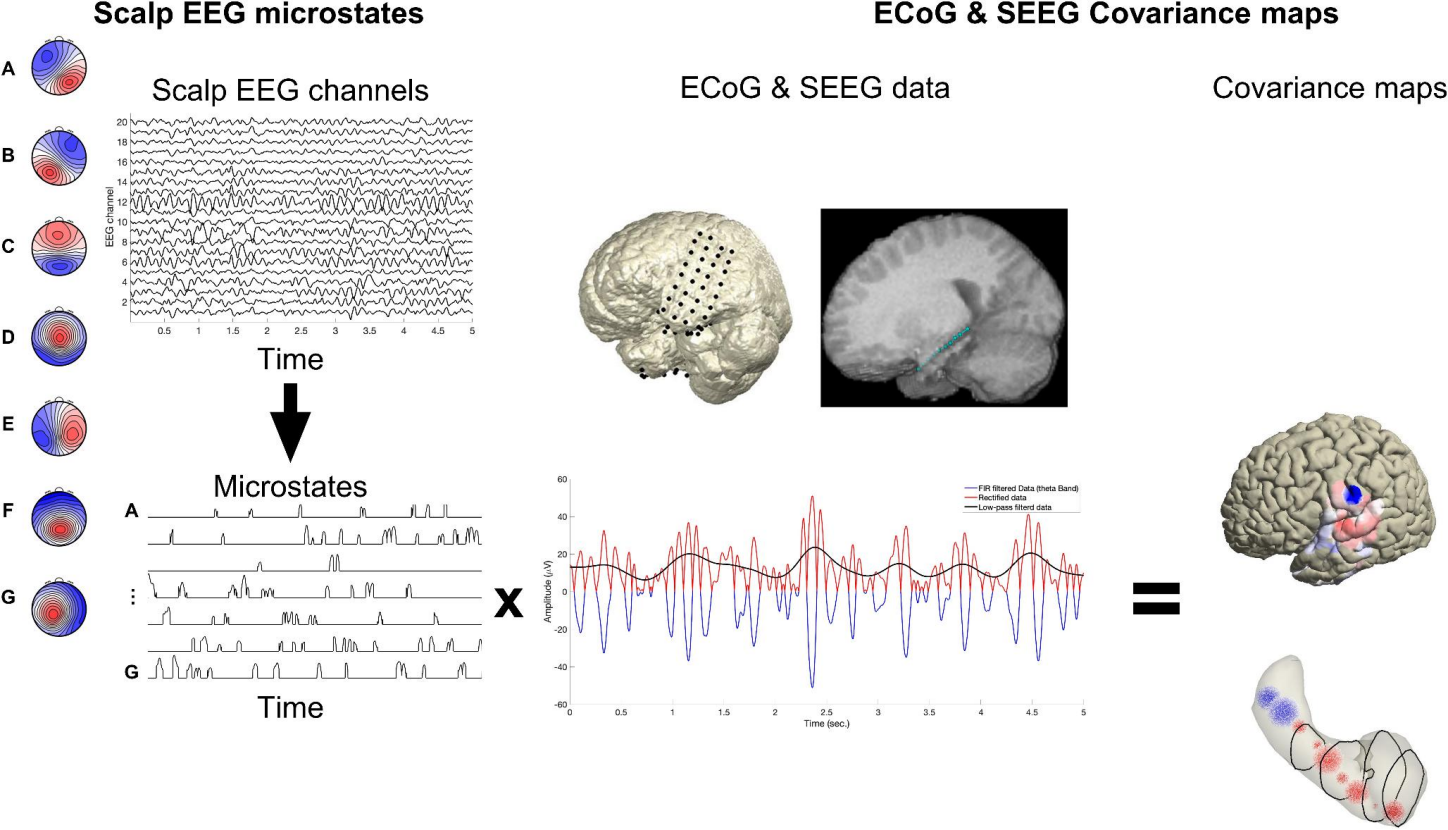
The outer left panel shows the seven microstate template maps of Custo et al. (2017). These microstate template maps were fitted on the resting-state scalp electroencephalography (EEG) data for each timeframe (winner takes all). We obtained seven timelines for the presence and strength of the different microstate classes over time (lower left panel). Electrocorticography (ECoG) and stereotactic electroencephalography (SEEG) results were recorded using the grid and depth electrodes (presurgery magnetic resonance imaging [MRI] results fused with postsurgery MRI results of participant 1; upper middle panel) were first filtered in the respective frequency band (blue; partially covered line in the lower middle panel), rectified (red line), and low-pass-filtered (black line), thus yielding the temporo-spectral evolutions of each intracranial channel. Finally, covariance maps were obtained by computing a weighted sum over these temporo-spectral evolutions using the microstate time series as weights. An example of the covariance maps of the alpha band in microstate class C of participant 1 is shown (right panel). FIR, finite impulse response

For each ECoG and SEEG channel, the covariance of the temporo-spectral evolution with the timeline of a given microstate class was computed as the mean of that temporo-spectral evolution weighted by the value of the given microstate timeline at the same moment. Epochs with artifacts were excluded. Such covariance analyses were performed for each participant, yielding separate distributions of each participant’s beta values (covariance maps) for each frequency band and microstate class for the ECoG and SEEG data. The details of this covariance analysis are provided elsewhere (Koenig et al. 2008; Mikutta, Altorfer, Strik, & Koenig, 2012). The significance of these covariance maps was tested using a permutation test with 1000 iterations, shuffling the temporal associations between the spectral amplitude dynamics and microstate timelines (TCT Reference). To get an estimate of the strength of the associations of the obtained covariance maps with the microstate timelines, the resulting covariance maps were back-projected onto the corresponding temporo-spectral evolutions of the ECoG/SEEG data, yielding a single time course for each covariance map. Then, these time courses were correlated with the corresponding microstate timeline to estimate the R-value. Only results with R values more than 0.1 were presented (Fig. 2).

### Cluster Analysis

To test how well the microstate maps of the two participants corresponded to the microstate templates of Custo et al. (Custo et al. 2017), we applied standard polarity invariant k-means clustering (where k was set to 7) to the maps at the momentary peaks of the global field power of each of the participants and calculated the correlation coefficients of the resulting individual microstate maps and the templates.

## Results

We estimated the scalp EEG-based microstate patterns over time by applying the seven templates of Custo et al. (Custo et al. 2017). Then, we performed a covariance analysis with the temporal dynamics of these scalp EEG-based microstates and the temporospatial evolution of ECoG and SEEG activity as input (Thut et al. 2006).

**Fig. 3:**
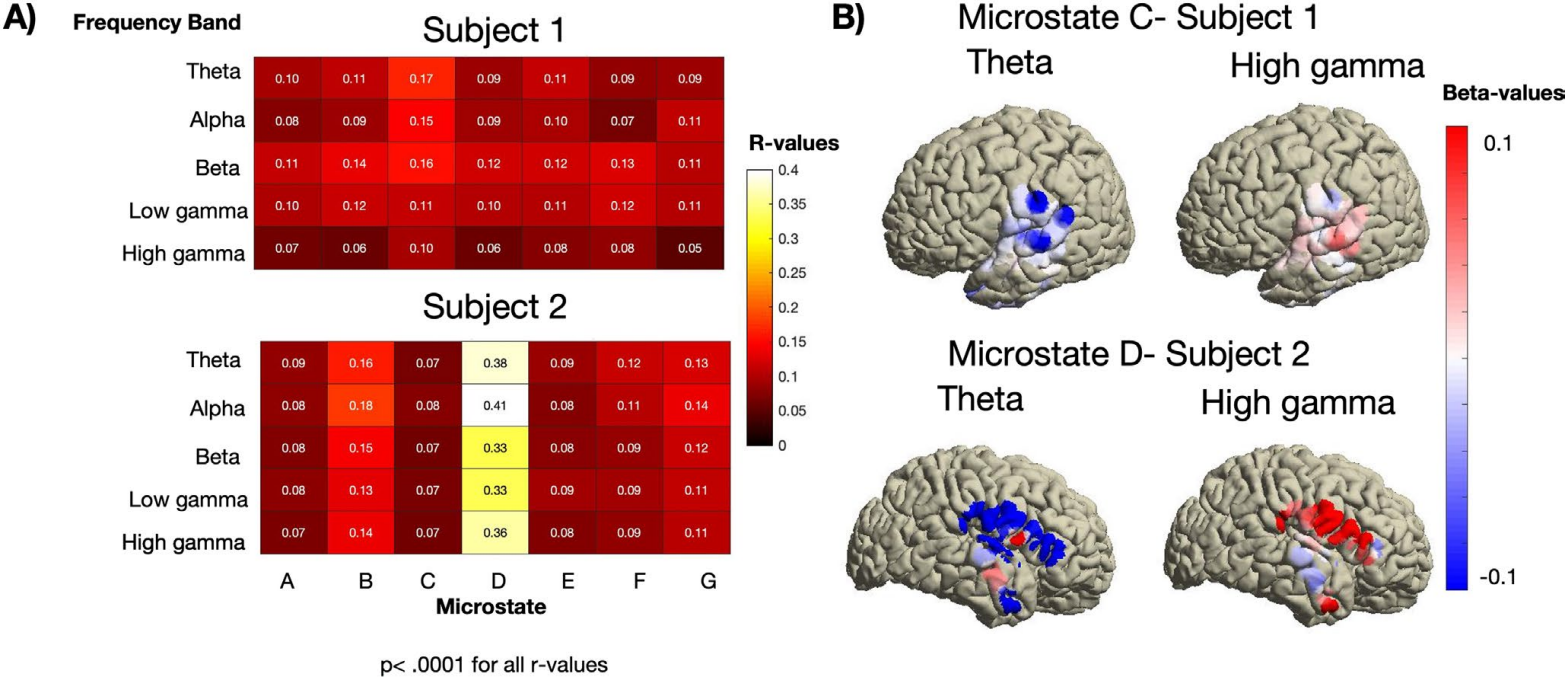
(a) The R-values as a function of the microstate class and frequency band of both participants obtained using electrocorticography (ECoG) electrodes. The highest correlations were found in microstate class C and microstate class D for participant 1 and participant 2, respectively. (b) Covariance maps localized to the left and right hemisphere. The upper panel shows the covariance maps of theta (left) and broadband high-gamma (right) in microstate class C for participant (1) The lower panel shows the covariance maps of theta and high-gamma in microstate class D for participant (2) Note the inverse relation of the two frequency bands. Supplementary Fig. 2 to 5 show the results of the other frequency bands

We found significant covariance maps for all seven microstate classes for all frequency bands (p < 0.0001, permutation test) for both participants using the ECoG electrodes (Fig. 3a). Furthermore, we found the highest correlation coefficients in microstate class C for participant 1 and microstate class D for participant 2.

Covariance map values at the corresponding ECoG electrodes identified distinct anatomical regions with positive and negative beta values associated with the presence of particular microstate classes. Among the largest beta values were those for microstate class C, localized in the left Heschl’s gyrus and the superior temporal gyrus of participant 1. The left medial temporal gyrus and inferior temporal gyrus also showed smaller beta values. Furthermore, we found large beta values in the right posterior inferior temporal gyrus at higher frequencies (beta, low-gamma, and high-gamma). The activation patterns of low-frequency bands (theta, alpha) and the high-gamma frequency band were inverse, indicating that negative beta values in the lower-frequency band were associated with positive beta values in the high-gamma band (upper panel of Fig. 3b and Supplementary Figs. 2 and 3).

The beta values associated with microstate class D were largest in the supramarginal gyrus, lower part of the somatosensory gyrus, and lower part of the frontal gyrus of participant 2. Furthermore, we found large beta values covering the precuneus at the midline (lower panel of Fig. 3b and Supplementary Figs. 4–6). We found the same inverse relationship between the beta values in low frequencies (theta and alpha) and broadband high-gamma in participant 2 (lower right panel of Fig. 3b).

In general the inverse relationship between the lower-frequency bands (theta, alpha) and the high-gamma frequency was found to be widespread across all microstates.

Regarding the SEEG electrodes covering the hippocampus and amygdala, we observed significant results for all frequency bands and microstate classes; however, the R values were reasonably large (> 0.1) only in microstate class D (Supplementary Fig. 1a and 1b).

Finally, we collapsed the covariance maps of the two participants into target regions of interest. The electrodes for different regions of interest were identified using BioImage Suite (Papademetris, 1993) Fig. 4 shows the mean beta values for all microstate classes of theta and broadband high-gamma activity as a function of these regions of interest. Again, we observed an inverse relationship between theta and high-gamma associated with microstate class D.

**Fig. 4:**
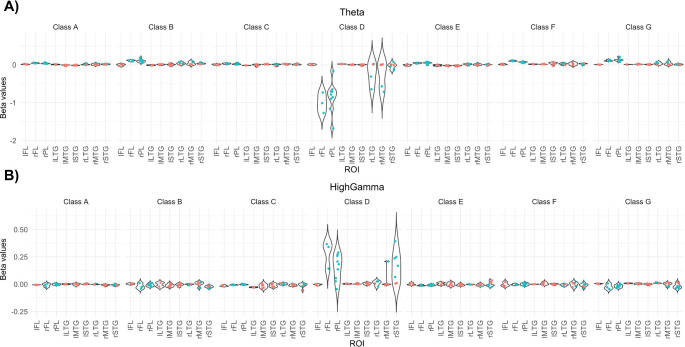
Mean beta values of key anatomical regions of the theta (upper panel) and broadband high-gamma (lower panel) bands of both participants (participant 1: red; participant 2: blue). Note the inverse relationship between the theta and broadband high-gamma bands in microstate class D. IFL, ; ILTG, ; IMTG, ; ISTG, ; rFL, ; rLTG, ; rMTG, ; ROI, region of interest; rPL, ; rSTG,

Additionally, we performed a cluster analysis of scalp EEG results. The microstate maps are shown in Supplementary Fig. 8. These maps appeared reasonably similar to the template maps of Custo et al., with mean correlations of r = 0.80 (participant 1) and r = 0.86 (participant 2).

## Discussion

We demonstrated that global scalp EEG-based microstate dynamics were systematically associated with local frequency-domain changes in intracranial activity, including broadband gamma activity. By combining scalp EEG microstate analysis with the temporo-spectral evolution of ECoG/SEEG local field potential data, we developed a method that potentially reduces the technical limitations of previous studies EEG-fMRI and inverse solution-based EEG studies that did not allow for or make use of a frequency-resolved analysis. To correlate scalp EEG-based microstate dynamics with ECoG/SEEG signals, our method used microstate activation timelines as regressors for the temporo-spectral evolution of ECoG/SEEG local field potentials. Therefore, we were able to capture the fast nature of microstate class changes with millisecond resolution and expand our analysis to broadband high-gamma bands, which reflect local neuronal population activity with high spatial resolution (Leszczynski et al. 2020).

Our method enabled us to correlate local field potential data derived directly from cortical electrodes with scalp EEG-based microstates at the same temporal resolution. Therefore, the method could potentially further disentangle the interactions between local activations/deactivations and global microstates. Furthermore, it could provide a basis for future projects exploring ECoG-based connectivity and EEG-based microstates.

We successfully demonstrated distinct activation patterns of the frequency-domain ECoG and (to a lesser degree) SEEG local field potentials associated with the concurrent presence of particular classes of global scalp EEG microstates. These associations were dependent on the frequency band, the location of the ECoG. The observation of such complex patterns of cortical activation and deactivation is in agreement with similar claims by recent studies of EEG microstates. The first study to investigate BOLD correlates of EEG microstates found that they were associated with both increases and decreases in BOLD signals (Britz et al. 2010). Deolindo et al. reported that a microstate class associated with the activation of the medial prefrontal cortex and attentional functions decreased when helicopter pilots conducted complex and potentially life-threatening maneuvers (Deolindo et al. 2021). Rajkumar et al. used a trimodal EEG, positron emission tomography, and fMRI design to explore the interaction of glutamatergic and gamma-aminobutyric acid (GABA)-ergic (GABAergic) neuroreceptor availability and the interaction with the EEG microstate. Glutamate is the main excitatory neurotransmitter, whereas GABA is the main inhibitory neurotransmitter. The results showed a strong correlation between GABAergic neuroreceptor availability and the EEG microstates. This indicates that the presence of a given microstate may also result in the deactivation of a given brain area (Rajkumar, Farrher, et al., 2021).

Our results also demonstrated that microstate-dependent activation/deactivation patterns were dependent on frequency bands, with broadband high-gamma activity showing an inverse relationship with lower frequencies. This might be particularly important because it is thought that microstates mediate local transient neural activity that is not necessary long enough to establish network-level coordination (Musso et al. 2010; Yuan, Zotev, Phillips, Drevets, & Bodurka, 2012). Therefore, broadband high-gamma activity might reflect short-time regional activations modulated by microstate patterns of the scalp.

Our findings of negative correlations between broadband high-gamma and low-frequency spectra are also in agreement with those of other studies that described decreased alpha power as a gateway for activation. This was observed with lower alpha activity, indicating higher excitability in the occipital visual cortex (Romei et al. 2008). Similarly, decreased alpha activity indicated heightened responsiveness to auditory stimuli and increased broadband high-gamma power in the superior temporal cortex (Potes et al. 2014). However, the correlation between microstate dynamics and lower-frequency (e.g., alpha) bands of the ECoG/SEEG electrodes might reflect modulations of long-range functional connectivity, as postulated by the CTC framework (Fries 2005).

The limited intracranial data allow only a partial comparison of the ECoG spectral correlates of the microstates with previous fMRI data. Custo et al. (2017) observed activation in the right inferior parietal lobe and the right middle and superior frontal gyri in microstate class D. These results resemble those of our study, which indicated that right ventral frontal negative activation is associated with this microstate class. Britz et al. (2010) performed a combined EEG-fMRI study and found a negative BOLD response in the right-lateralized dorsal and ventral areas of the frontal and parietal cortices in microstate class D. Furthermore, using minimum norm source estimation of a wideband EEG signal (4–30 Hz), larger-scale anatomical regions of activation and deactivation were observed, similar to our results (Coquelet et al. 2021). During this analysis, deactivation in the parieto-frontal and temporal regions was observed in microstate class D, which was in accordance with our results indicating deactivation in parieto-frontal ECoG channels at lower frequencies.

This study had some limitations. First, it included a limited number of ECoG/SEEG electrode positions (44 and 85 electrodes). Another limitation of our study was the possible alterations in brain activity attributable to epilepsy, even though epileptic activity was excluded via automated detection processes. Recent studies have argued that the epilepsy-affected brain can be used as a proxy for the normal human brain (Parvizi and Kastner 2018). Moreover, the clinical setting may have interfered with normal resting-state measurements.

In summary, we have provided initial evidence indicating that global scalp EEG microstates are correlated with ECoG/SEEG field potentials. These correlations span a broad range of frequency bands with systematic inverse relationships between lower and higher bands. These results emphasize the notion of complex self-organization, even in the absence of concrete external stimuli (Fox and Raichle 2007).

### Electronic Supplementary Material

Below is the link to the electronic supplementary material.

Supplementary Material 1

Supplementary Material 2

Supplementary Material 3

Supplementary Material 4

Supplementary Material 5

Supplementary Material 6

Supplementary Material 7

Supplementary Material 8

## Data Availability

The datasets generated during this study are available from the corresponding author upon reasonable request.
