# Supplementary figures and images for: Electrocorticographic Activation Patterns of Electroencephalographic Microstates
Source: Brain Topogr. 2023 Mar 20;37(2):287–95. doi: 10.1007/s10548-023-00952-1 (PMC10884069; doi:10.1007/s10548-023-00952-1)

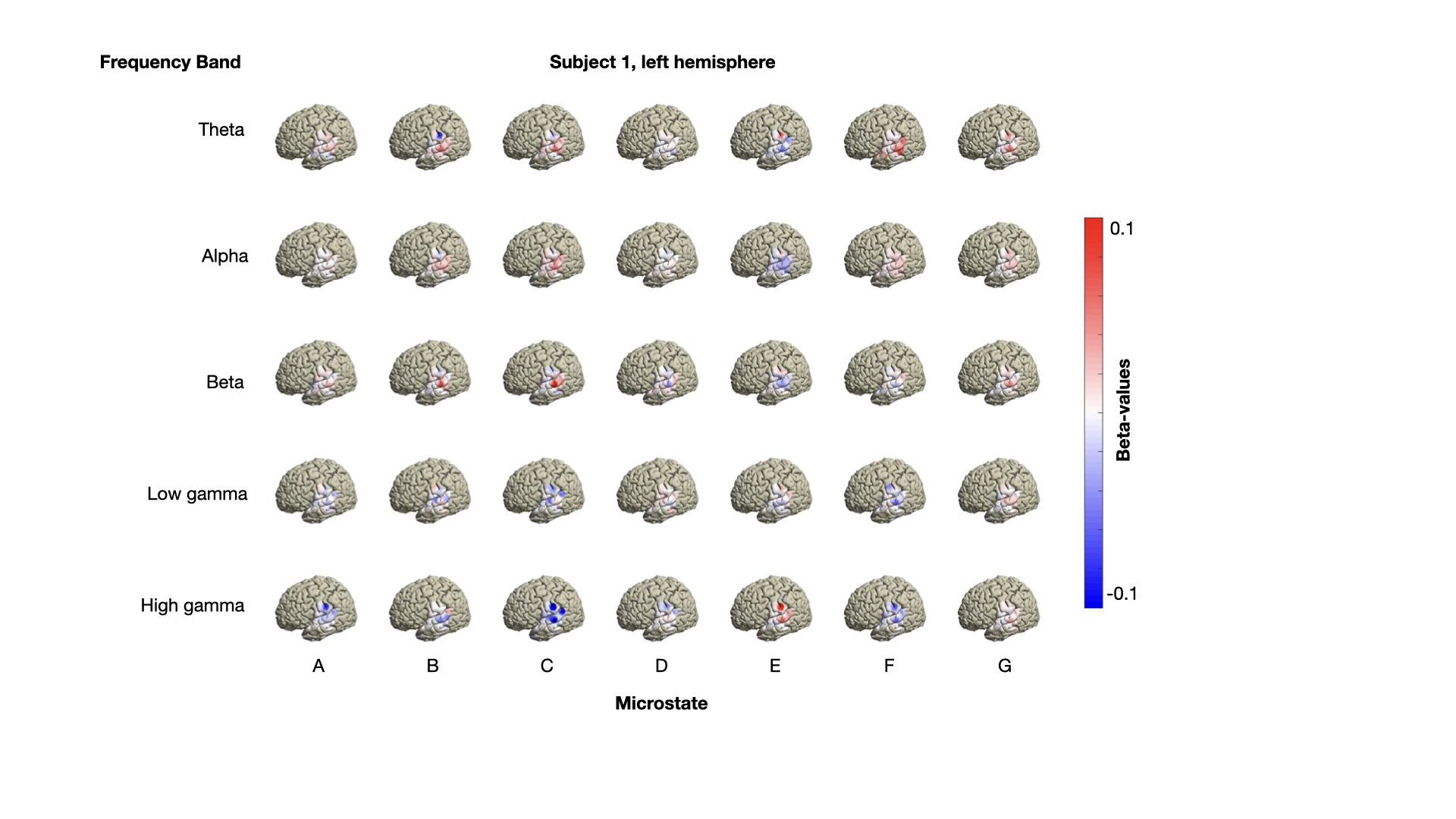

Supplement: Supplementary file 1 — Supplementary Material 1 [file 10548_2023_952_MOESM1_ESM.tiff]

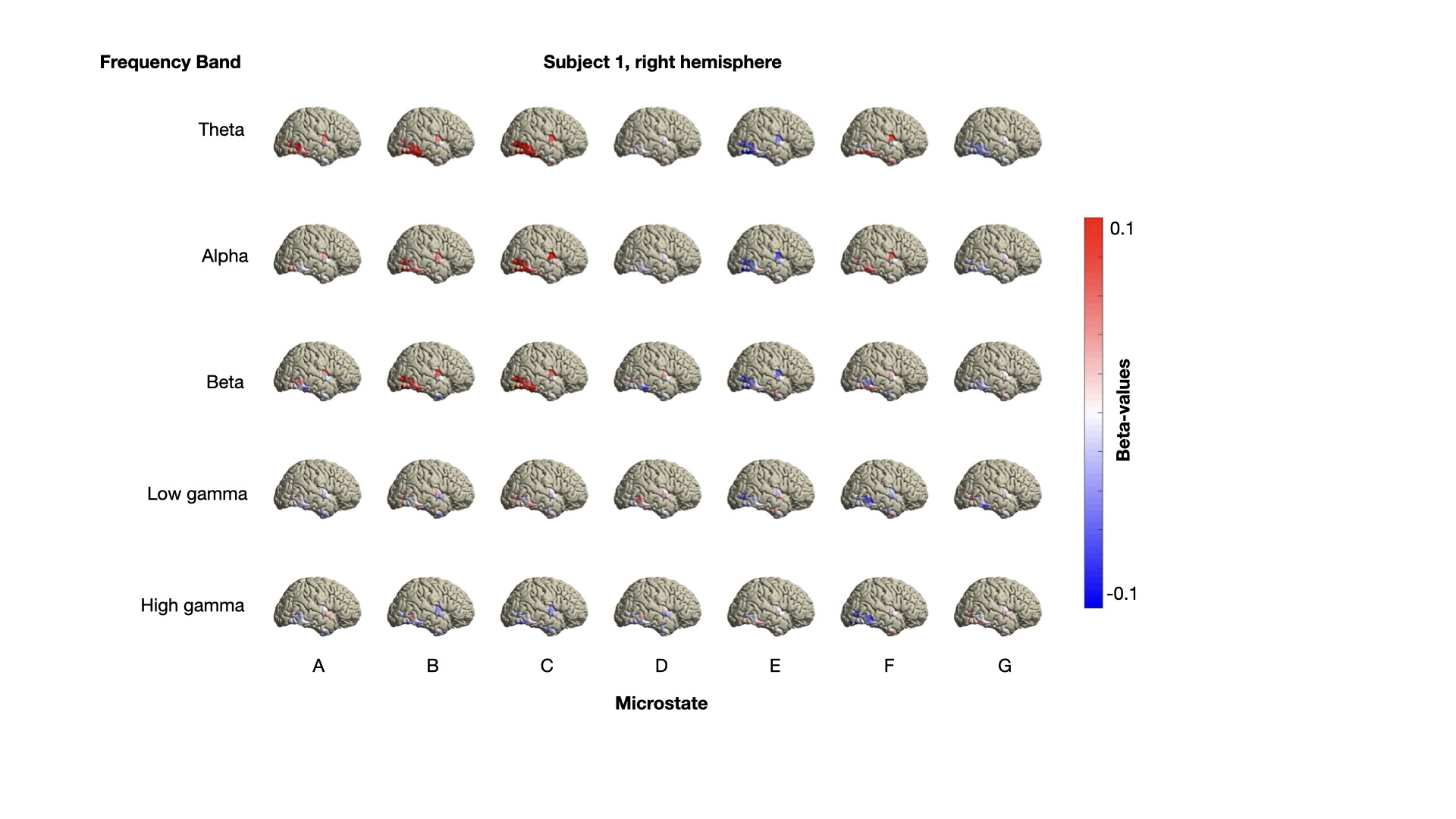

Supplement: Supplementary file 2 — Supplementary Material 2 [file 10548_2023_952_MOESM2_ESM.tiff]

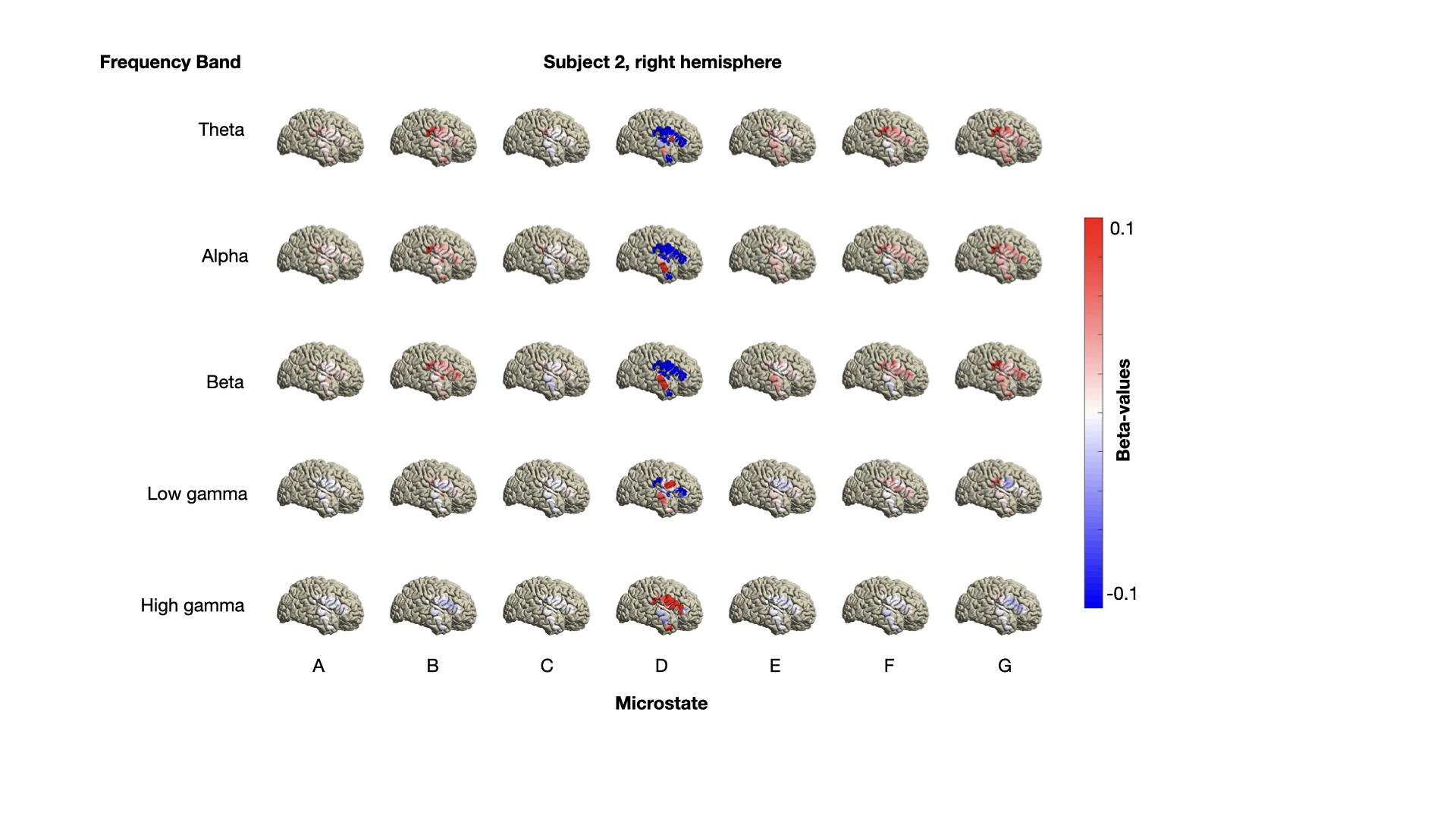

Supplement: Supplementary file 3 — Supplementary Material 3 [file 10548_2023_952_MOESM3_ESM.tiff]

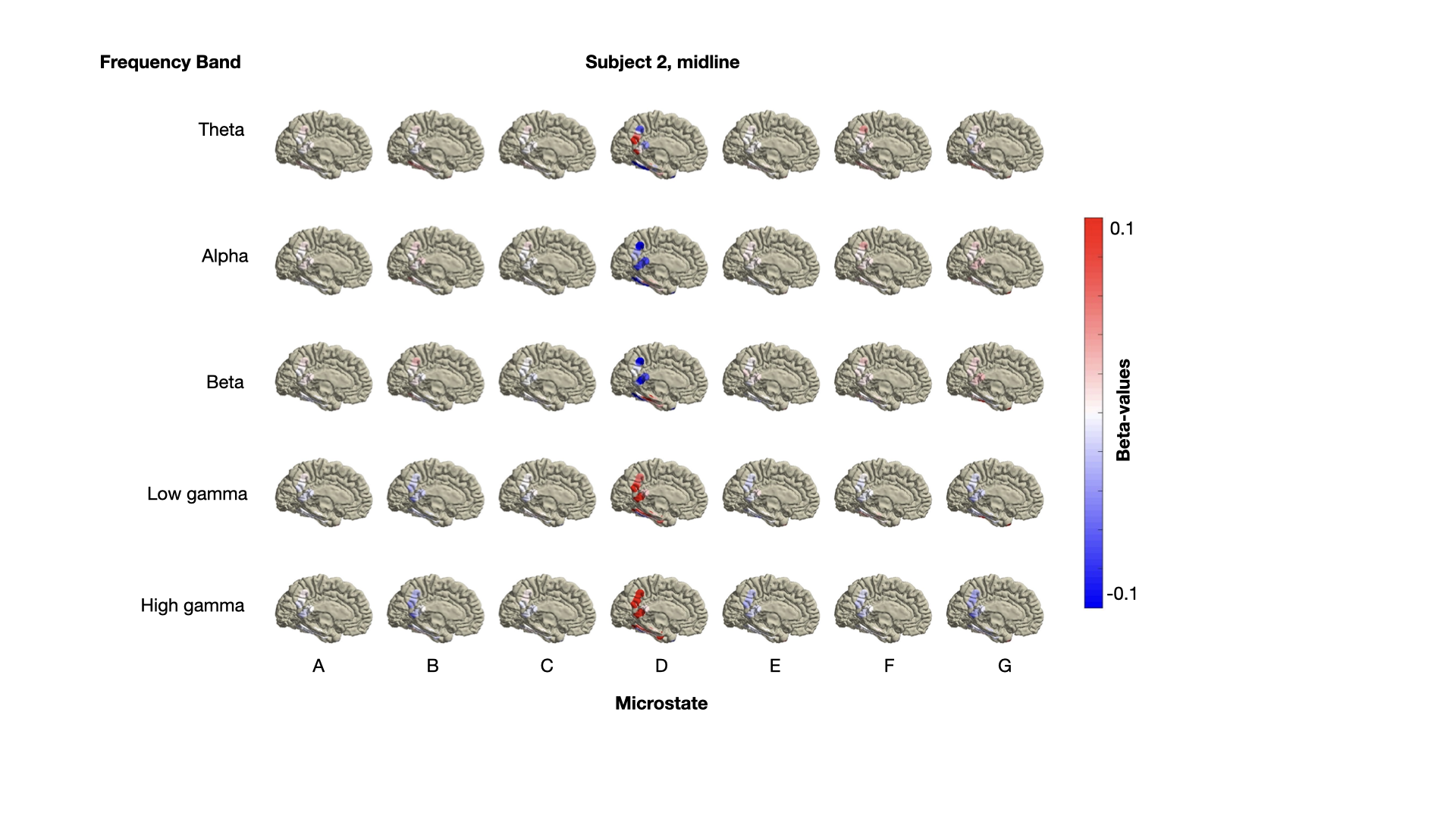

Supplement: Supplementary file 4 — Supplementary Material 4 [file 10548_2023_952_MOESM4_ESM.tiff]

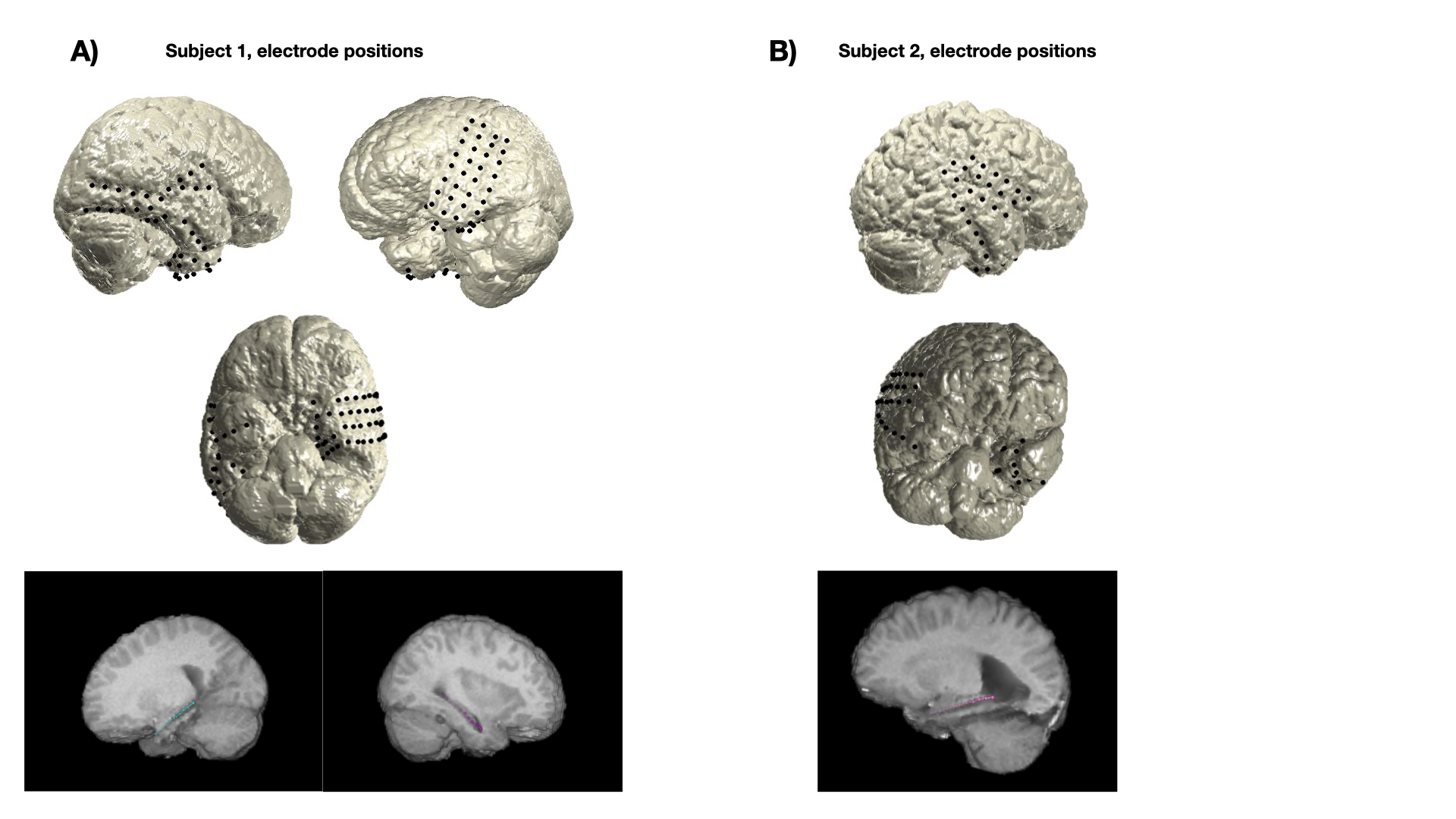

Supplement: Supplementary file 5 — Supplementary Material 5 [file 10548_2023_952_MOESM5_ESM.tiff]

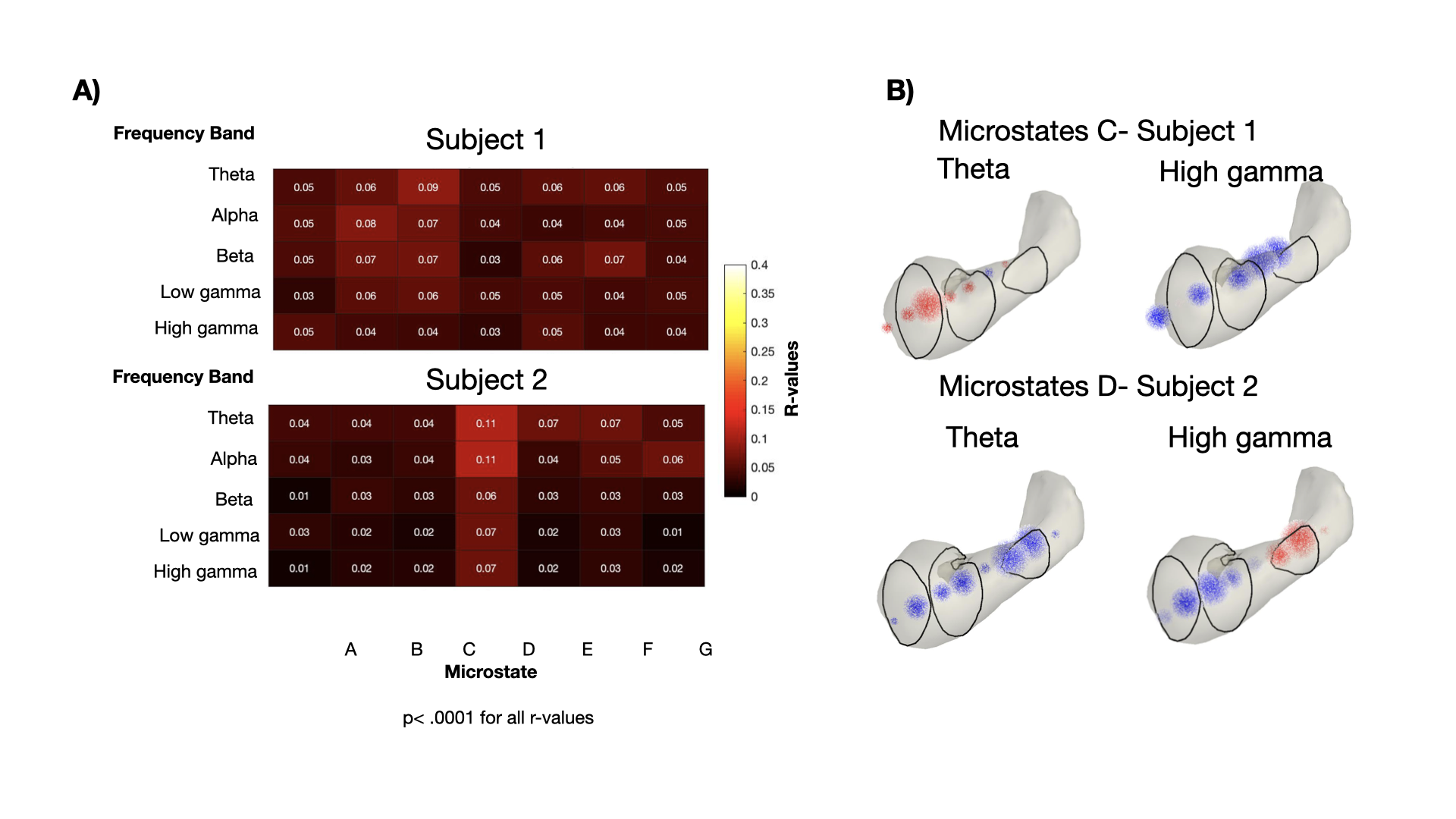

Supplement: Supplementary file 6 — Supplementary Material 6 [file 10548_2023_952_MOESM6_ESM.tiff]

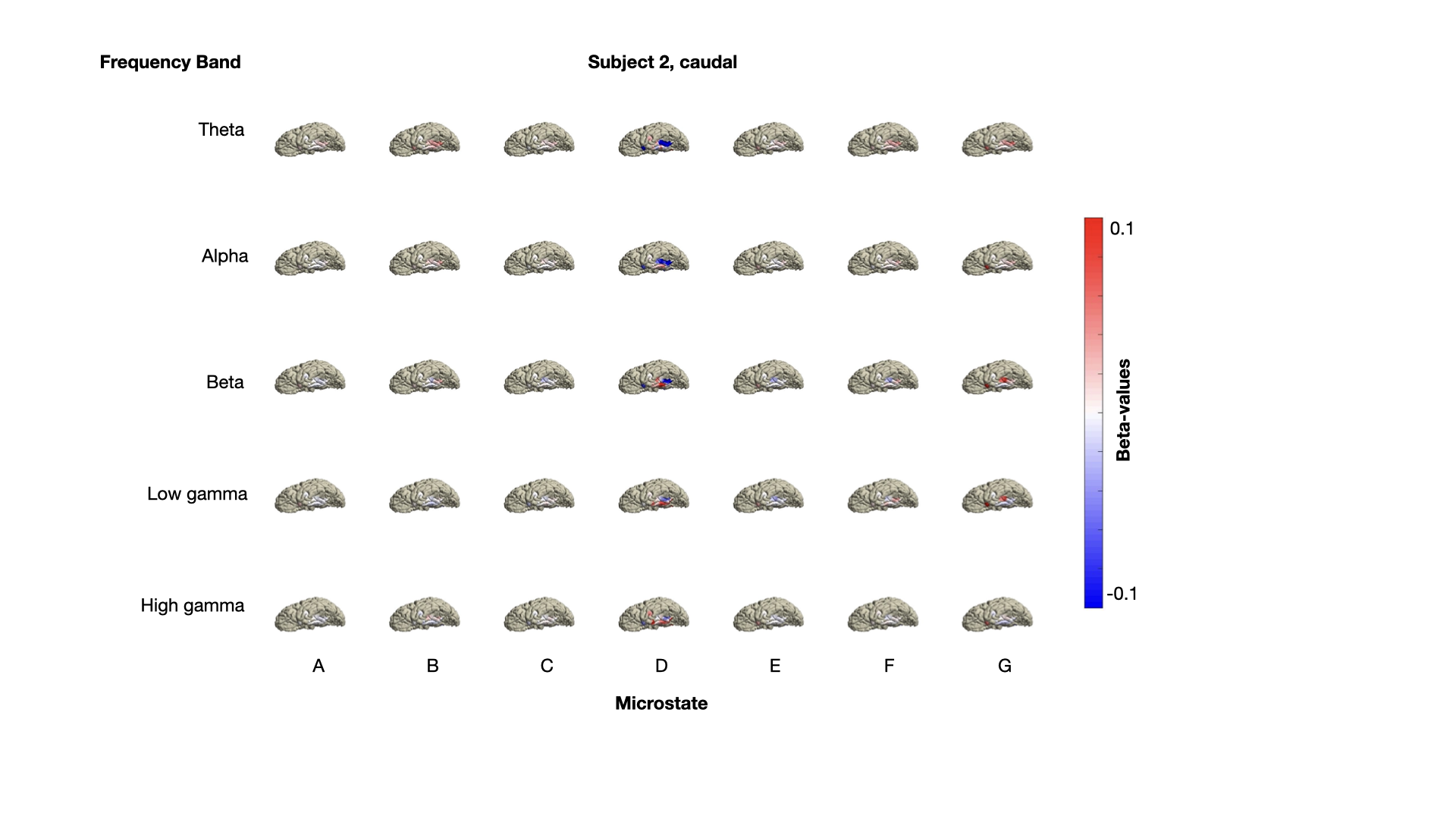

Supplement: Supplementary file 7 — Supplementary Material 7 [file 10548_2023_952_MOESM7_ESM.tiff]
