# Supplementary material for: Electrocorticographic Activation Patterns of Electroencephalographic Microstates
Source: Brain Topogr. 2023 Mar 20;37(2):287–95. doi: 10.1007/s10548-023-00952-1 (PMC10884069; doi:10.1007/s10548-023-00952-1)
Supplement: Supplementary file 8 — Supplementary Material 8 [file 10548_2023_952_MOESM8_ESM.docx]

**Supplementary Figure Captions**

**Supplementary Fig. 1** (a) R values as a function of the microstate class and frequency band of both participants using stereotactic electroencephalography (SEEG) electrodes. Similar to the electrocorticography (ECoG) data, the highest correlations were found in microstate class C and microstate class D for participant 1 and participant 2, respectively. Although all microstate classes and frequency band combinations were significant, only microstate class C (participant 1) and microstate class D revealed meaningful R values (>0.09). (b) Beta values of SEEG electrodes covering the hippocampus and amygdala. The theta (left) and high-gamma (right) beta values in microstate class C for participant 1 (upper panel) are shown. The theta and broadband high-gamma activations in microstate class D for participant 2 (lower panel) are shown

**Supplementary Fig. 2** Beta values of the left hemisphere (participant 1). Rows indicate frequency bands (theta, alpha, beta, low-gamma, high-gamma). Columns indicate microstate classes A to G

**Supplementary Fig. 3** Beta values of the right hemisphere (participant 1). Rows indicate frequency bands (theta, alpha, beta, low-gamma, high-gamma). Columns indicate microstate classes A to G

**Supplementary Fig 4.** Beta values of the right hemisphere (participant 2). Rows indicate frequency bands (theta, alpha, beta, low-gamma, high-gamma). Columns indicate microstate classes A to G

**Supplementary Fig. 5** Beta values of the medial left hemisphere (participant 2). Rows indicate frequency bands (theta, alpha, beta, low-gamma, high-gamma). Columns indicate microstate classes A to G

**Supplementary Fig. 6** Beta values of the caudal right temporal lobe (participant 2). Rows indicate frequency bands (theta, alpha, beta, low-gamma, high-gamma). Columns indicate microstate classes A to G

**Supplementary Fig. 7** Electrode positions of the two participants. (a) Participant 1 had 78 electrocorticography (ECoG) and 20 stereotactic electroencephalography (SEEG) electrodes. ECoG electrodes covered the lateral left and right parts of the temporal lobes. SEEG electrodes covered the left and right hippocampus and amygdala. (b) Participant 2 had 52 ECoG and 10 SEEG electrodes. ECoG electrodes covered the right parietal, frontal, and temporal areas. SEEG electrodes covered the left hippocampus and amygdala

**Supplementary Fig. 8** Seven microstate templates identified by Custo et al (2017) are shown (upper row). The middle row shows the real data microstate maps of participant 1. The bottom row depicts the real data microstate maps of participant 2. Note the similar microstate patterns of both participants compared to those of the templates
